# The role of institutional ethics committees in Austria: Report of the Commission on Ethics and Scientific Integrity of the Karl Landsteiner University of Health Sciences 2018–2023

**DOI:** 10.1007/s00508-024-02462-x

**Published:** 2024-10-10

**Authors:** Sophie Schober, Sascha Klee, Franz Trautinger

**Affiliations:** 1 Commission on Ethics and Scientific Integrity, Karl Landsteiner University of Health Sciences, Dr.-Karl-Dorrek-Straße 30, 3500 Krems, Austria; 2 Institute of Science and Technology (ISTA), Am Campus 1, 3400 Klosterneuburg, Austria; 3 Division of Dermatology, University Hospital St. Pölten, St. Pölten, Austria; 4 Division of Biostatistics and Data Science, Karl Landsteiner University of Health Sciences, Krems, Austria

**Keywords:** Research ethics, Clinical research, Informed consent, Good scientific practice, Study participants

## Abstract

Research involving human subjects or identifiable human material and data must be assessed by an ethics committee. The Karl Landsteiner University of Health Sciences has established a Commission on Ethics and Scientific Integrity to evaluate medical research conducted by its faculty and students and at its affiliated hospitals.

All projects submitted to the Commission on Ethics and Scientific Integrity between 2018 and 2023 were analyzed regarding their major characteristics, the duration of the evaluation process, and votes issued.

A total of 520 applications were electronically submitted during the observation period. Most of the studies were retrospective data analyses in the field of oncology, psychology and surgery. Most studies included less than 100 volunteers. Of the applications 50% received a final vote within 5 months, during which several revision rounds took place. Overall, about 77% of votes issued during the observation period were positive and 2% were rejections. In 11% files were closed due to withdrawal. In 11% final votes were pending at the end of the observation period due to requests for revisions.

Our results emphasize the importance of institutional ethics committees using the example of the Commission on Ethics and Scientific Integrity at the Karl Landsteiner University. Such committees fill a gap in evaluating research not covered by Austrian legal regulations. Continuous development of standards, operating procedures, and national and international collaborations are required to assess and minimize risks to trial subjects and to provide a safe and productive environment for research in human medicine and related fields.

## Introduction

Ethics committees (ECs) are (mostly interdisciplinary) boards that are independent (*weisungsfrei*) and primarily examine and critically review research involving human subjects [1]. The work of Austrian ECs is based on ethical principles and international standards as set out in the Declaration of Helsinki (2022), the International Council for Harmonisation (ICH) guidelines for good clinical practice E6(R2) (2002) as well as the Austrian Medicinal Products Act (AMG), the Austrian Medical Devices Act (MPG), the hospital laws of the federal states (in our case the *Krankenanstaltengesetz* of Lower Austria, NÖ KAG) and the Federal Act on Hospitals and Health Resorts (KAKuG). Additionally, national laws that have to be considered are the Genetic Engineering Act (GTG), the Research Organization Act (FOG), and the Data Protection Act (DSG), the latter referring to the General Data Protection Regulation of the European Union (GDPR) [2].

In accordance with the KAKuG and the Universities Act (UG), in addition to the mandatory and legally defined ECs in hospitals and public medical universities, private universities, universities of applied sciences, and other institutions involved with research in humans have voluntarily established institutional ECs or ethics advisory boards (institutional review boards, IRBs). Without a legal framework their remit and composition are regulated autonomously in the statutes of the respective university reflecting its research orientation [1]. Thus, they must be distinguished from the abovementioned mandatory ECs and from other institutions for the protection of scientific integrity [3].

The Karl Landsteiner University of Health Sciences (KL) is a private university founded in 2013 with study programs in medical science, human medicine, and psychology. In accordance with the university’s statutes (https://www.kl.ac.at/en/satzung) the Commission on Ethics and Scientific Integrity of the Karl Landsteiner University of Health Sciences (KL-EC, https://www.kl.ac.at/en/research/ethics-committee) was established in 2016. It consists of eight members and their deputies namely one representative of the university’s academic staff and senate, an external lawyer, an external medical expert, an external natural scientist, a medical ethics specialist, a statistician and a clinical psychologist as well as an administrative officer. The KL-EC assesses research projects that require EC approval carried out at the university and its affiliated hospitals or by investigators from these institutions. Studies that fall under the AMG or the MPG must be assessed by the abovementioned statutory committees. Otherwise, following the principles of the Helsinki Declaration, all “medical research involving human subjects, including research on identifiable human material and data” must be approved by the KL-EC before conduction [4]. Exemptions have been defined for anonymized studies using residual materials from examinations, procedures performed as part of routine medical care (medical waste) and for anonymized (mainly questionnaire) studies that do not include patients or subjects from vulnerable groups and that do not collect health-related data. The general objectives (i) protection of rights and welfare of volunteers and patients, (ii) support for researchers, and (iii) transparency for the public as described in the literature [5, 6], equally apply to the KL-EC. To this end, assessments of ECs include: (i) the qualification of the investigator, (ii) the available facilities and staff, (iii) the scientific validity of the study protocol and its risk-benefit ratio, (iv) the recruitment of participants and type and form of informed consent, and (v) provisions regarding insurance, that may be required [5]. All approved projects are publicly available. At least the following documents must be submitted: application form, study protocol, confidentiality declaration, disclosure of conflicts of interest and curriculum vitae of the principal investigator. Additional documents to be submitted, if necessary: patient recruitment material, information and consent form, confirmation of insurance, questionnaires, and other supplementary material (e.g., case record forms) and votes already issued for the study from other ECs/IRBs. After formal review, accepted projects are assessed by the (deputy) members of the commission. Once a month, meetings are held to issue votes based on these evaluations. Possible outcomes are: “withdrawn”, “rejected”, “postponed”, “provisionally approved”, and “approved”. Projects can also be withdrawn by the applicant at any time. “Provisionally approved” projects can receive approval as soon as appropriate revisions have been received (without the need of further assessment by the whole committee). Postponed projects (requiring major revisions) will be re-evaluated by the committee members as described above. All other notifications (amendments and renewals) are processed on an ongoing basis.

As the functioning and relevance of the work of so-called voluntary ethics committees in Austria have not been publicly documented so far, the aim of this paper is to describe the activities of the KL-EC between 2018 (the year in which an online submission and evaluation system was introduced) and 2023 to illustrate the relevance of voluntary ethics committees in Austria.

## Material and methods

All projects submitted to the KL-EC via the KL’s online submission system (ECS) between 1 January 2018 and 31 December 2023, are included in this evaluation. The ECS is a web application for handling ethics applications developed by a collaboration of the Medical Universities of Vienna and Innsbruck and is used by ECs throughout Austria. The total number of submissions, the number of submissions that were formally approved, evaluated, and dealt with in a meeting, the votes issued after the initial review, how many submissions ultimately received a positive vote, and the time span from submission to a possible start of the study (= issue of a positive vote) were analyzed. Furthermore, to provide an overview of the content of the projects, the allocation to medical subject categories and the types of the studies are summarized. The planned number of trial participants (sample size) was categorized (< 100, 100–499, 500–999, ≥ 1000) and analyzed using descriptive methods (bar charts, pie charts, mean/median values and relative ratios). All data were saved and analyzed using Microsoft®Excel® 2016 (© 2016 Microsoft Corporation, Redmond, USA). The evaluation of the data was completely anonymized so that no traceability to individual submitters, investigators, projects, and study participants is possible.

## Results

**Fig. 1:**
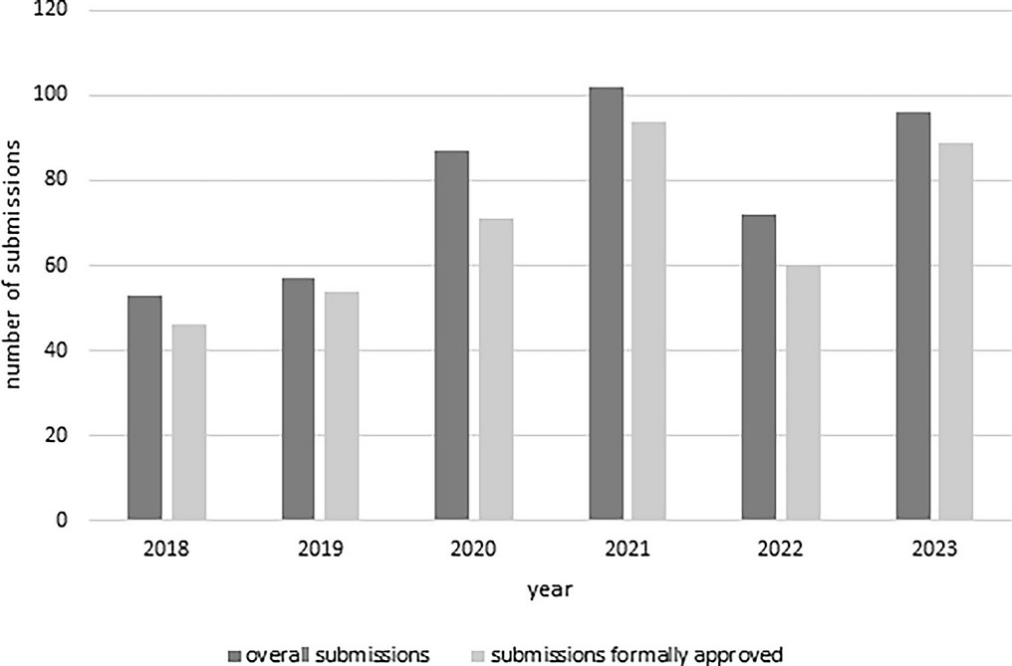
Yearly submissions: Overall, 467 complete applications were submitted to the KL-EC between 2018 and 2023. Approximately 80–95% of all studies could be formal approved. Approximately 5–20% of all studies submitted each year are still not formally approved (including studies which do not need an ethics vote, which are not within the remit of the KL-EC or are not resubmitted after being initially rejected during the formal review due to deficiencies)

The total number of applications submitted during the analysis period was 520, with 450 votes issued and 70 projects pending at the cut-off date, corresponding to an average of 87 applications and 75 votes per year. Of the committee meetings 64 were held with an average of 8 new applications per meeting (range 5–24) with January being the month with the lowest number of applications per meeting. The overall formal approval rate was 89% (414 out of 467) with yearly variations as shown in Fig. 1. The highest number of submissions was recorded in 2021 (102), of which 94 (approx. 92%) received formal approval. Negative formal reviews were, in addition to incomplete applications, mainly due to evidence that the submitted study does not fall within the remits of the KL-EC for reasons as described above. Some projects, initially rejected on formal grounds (*n* = 53), were never resubmitted for unknown reasons.

**Fig. 2:**
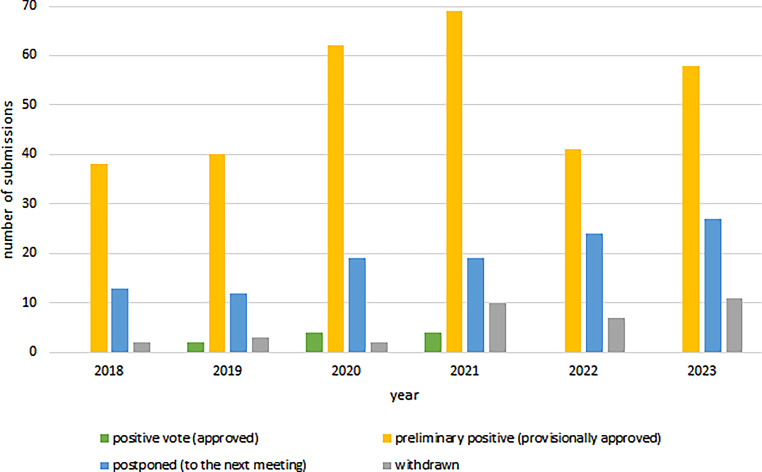
Number of initial decisions (positive vote, preliminary positive, postponed, withdrawn) of the KL-EC per year. In every year analyzed, the most frequent initial decision was “preliminary positive”

After initial evaluation, 10 (approx. 2%) positive and 308 (approx. 68%) preliminary positive votes (out of 450 evaluations) were issued. Of the initial applications 114 (approx. 25%) were postponed due to the need for major revision and 18 (4%) of the applications were withdrawn by the applicant. No project was rejected upon initial evaluation (Fig. 2). The overall number of votes and assessments is higher than the overall number of formal approvals, because some applications assessed as withdrawn have primarily not been formally approved.

After repeated assessment (*n* = 450 within the observation period), 346 (approx. 77%) positive votes (approved) were issued, 49 applications (approx. 11%) were withdrawn by the applicants and 7 applications (approx. 2%) were rejected by the KL-EC (overall number of final votes 402). The remaining applications (48; approx. 11%) had not been finally assessed at the cut off-date. The median duration of an evaluation from submission until a positive vote was 132.5 days (range 14–1656 days).

The majority of projects submitted were theses (322; 62%), 301 (approx. 58%) were retrospective studies and 18% were questionnaire/interview studies. Only a few applications (1–3%) were characterized as pilot studies, registries and biobanks (multiple allocations possible).

**Fig. 3:**
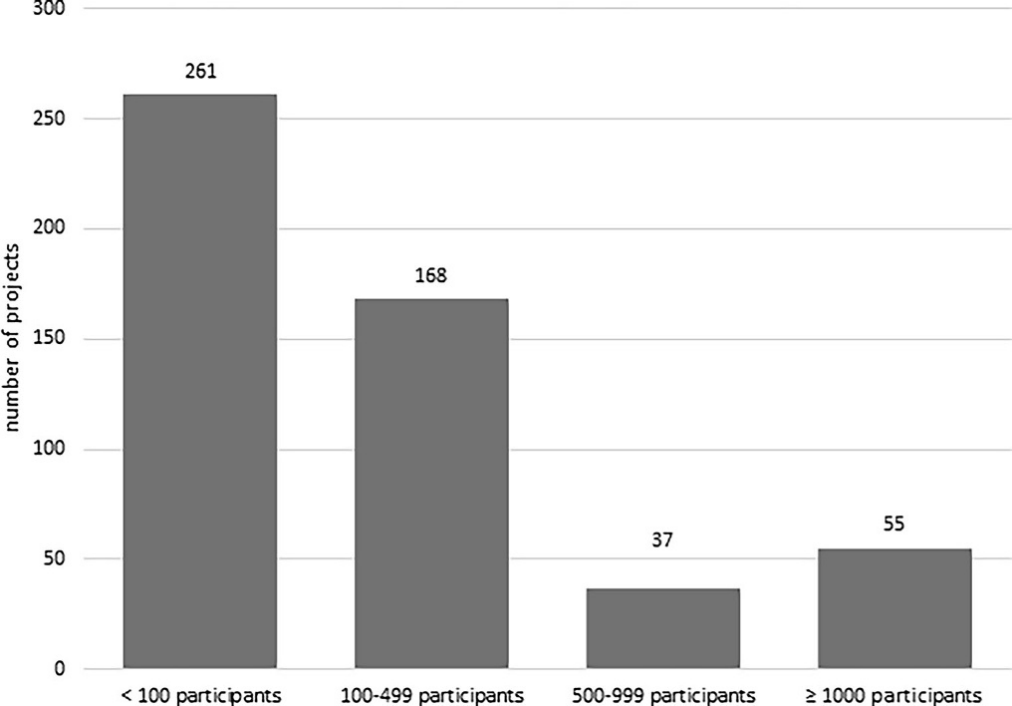
Distribution of projects according to the planned number of study participants (all years)

On average, submissions were topically allocated to up to three medical categories, as shown in Table 1. Apart from statistics, relevant to the majority of projects, medical specialties were broadly distributed within oncology, psychology and surgery being the top categories. In size, 261 studies planned to involve fewer than 100 subjects, 168 studies aimed at 100–499, 37 studies on 500–999 and 55 on 1000 or more than 1000 (Fig. 3). The minimum number of study participants was one (case report), the maximum number of study participants 25,000 (median: 100).

**Table 1:** Assignment of the submissions to the different medical categories (multiple answers possible)

Special field	Number of assignments
Statistics	416
Oncology	86
Psychology	66
Surgery (including pediatric, cardiothoracic and plastic surgery)	66
Intensive care/emergency medicine	42
Pharmacology (clinical)	40
Cardiology	39
Psychiatry	39
Infectiology and hygiene	38
Neurosurgery	35
Neurology	34
Gynecology	25
(Immuno)Pathology	24
Pediatrics	23
Public health	22
Orthopedics	20
Dermatology	19
Radiotherapy/radiology	17
Hematology	16
Child and adolescent psychiatry	15
Endocrinology	14
Nephrology	14
Anatomy	13
Laboratory medicine	12
Pulmonology	12
Gastroenterology	11
Anesthesia	10
Trauma surgery	10
Urology	10
Ophthalmology	7
Nutrition	6
Physical medicine	6
Health and healthcare	5
Angiology	3
Ear, nose and throat	3
Neonatology	2
Medical physics	1
Nuclear medicine	1
Pharmacy	1
Physiology	1
Virology	1

## Discussion

In accordance with its statutory obligations, the KL-EC ensures compliance with ethical standards in the conduct of research involving human subjects, human material and data at the KL and its affiliated university hospitals. The number of submissions highlights the need for institutional ECs and the large proportion of student theses illustrates that beyond its formal remit, the KL-EC, like other university review boards, also has an important educational and quality assurance task. We observed that only few applications pass the initial formal assessment without any queries, thus contributing to prolonged review periods. A reason for this might be that in a young university many submissions come from less experienced applicants and from medical students requiring support in the preparation of complete and assessable applications. Once formally approved, however, most projects receive provisionally positive votes, as only minor corrections or additions are required, which upon revision and resubmission by the authors, can be re-evaluated by the chairman and office alone, without the need to involve the whole committee.

Our analysis shows that corresponding to the KL’s curricula and the broad clinical scope of our affiliated hospitals, submissions cover a broad spectrum of topics. Due to the legal limitations of the commission described in the introduction, the distribution of study types is narrow and confined mainly to retrospective analyses and questionnaire studies.

In the following we provide an overview about specific challenges and issues faced by ECs. Some of these generally apply to all types of medical ethics committees and boards, others are specific for voluntary commissions, like the KL-EC.

As there is no regulation in Austria requiring special training for EC members, they are usually appointed according to their individual expertise and professional background as stipulated by law or in the statutes of the institution by the respective authorities (rectorates, governments). Thus, expertise in medical ethics varies, (particularly between institutional not legally regulated ECs) often resulting in divergent decisions on identical multicentric protocols when submitted to more than one EC. Currently, the standards of the ECs are so different that it usually must be decided on a case by case basis whether the vote of another EC is recognized or not [7]. National standardized mandatory and voluntary training programs would be useful to improve this problem [8, 9]. Once a common standard is established, a national agreement on mutual recognition of votes should follow, with the aim that common study protocols are maintained for national multicentric research projects, and only informed consent forms (ICF), other patient-related material and advertisements are site-specific. This does not apply to clinical trials according to AMG and MPG, where protocol integrity is already regulated by national and international stipulations.

Similarly, not only the expertise of ECs and individual EC members can vary but (as the results of our analysis show) also even more so the expertise of applicants, investigators and institutions. As the detrimental effects of this problem on medical progress, institutional performance, and individual careers are evident, it is the responsibility of ECs and their members to offer and participate in targeted training programs for current and future investigators. To this end, the KL not only offers relevant courses for students, but also training opportunities for scientific staff as part of its science skills services. These programs include courses on topics such as the ethical and legal framework of medical research, the basics of scientific methodology and statistics.

While the approval and conduct of clinical trials investigating medical products and devices is subject to strict regulations in Europe and worldwide, the ethical evaluation of other medical research is, at least in Austria, not or only insufficiently regulated. The § 19e (3a) of the NÖ-KAG for example states: “Vor der Durchführung angewandter medizinischer Forschung und von Pflegeforschungsprojekten und der Anwendung neuer Pflege- und Behandlungskonzepte und neuer Pflege- und Behandlungsmethoden kann die Ethikkommission befasst werden” (The ethics committee may be consulted before conducting applied medical research and nursing research projects and before applying new nursing and treatment concepts and new nursing and treatment methods). This appears to be in contrast with the Declaration of Helsinki that requests that medical research protocols “involving human subjects, including research on identifiable human material and data” must be submitted to “the research ethics committee concerned”. This ambiguity sometimes leaves researchers and ECs also in the dark about their obligation to submit and evaluate. It goes without saying that in cases of doubt and in the interest of participating patients and their material and data, the principles of the Declaration of Helsinki are paramount, and editors of scientific journals rightly require accompanying documentation of ethical approval along with the submission of manuscripts containing results of research in humans.

A frequent criticism on the need to apply for EC approval in medical research is that it is cumbersome and may delay research. Although it is obvious (and confirmed here also for the KL-EC) that approval may take time, this complaint disregards the fact that, similar to the peer-review process in scientific publishing, project review by a qualified EC leads to improvement of submitted protocols, thus preserving not only patient safety but at the same time increasing the scientific value of the proposed investigations. Formal enquiries often relate to the incorrect or incomplete completion of the online application form used by all ECs in Austria, which is designed as a standardized document for all types of studies. This makes the form appear complicated and confusing, especially to the inexperienced applicant. It is therefore the responsibility of all Austrian ECs to continuously develop this indispensable document in order to make it more convenient for the applicant and at the same time adapt it to a constantly evolving scientific landscape. In addition, while the document is primarily aimed at physicians conducting clinical trials, other disciplines conducting research on humans (e.g., psychology, nursing) often require EC approval for their projects. Both quantitative and qualitative research has to be evaluated. Therefore, mutual agreement to adapt the submission procedure and documents for these research areas and scientific methodologies is required as well. The question of whether separate or joint review committees and review procedures are preferable remains open [10].

While we tried to demonstrate and discuss issues relevant to EC submission and approval in Austria, our analysis is limited by the fact that it is primarily based on the experience, performance, and activities of a single institutional EC at a private university and thus might not be representative. Furthermore, a major part of clinical research, namely clinical trials of medical products and devices, could not be considered in our analysis and discussion as it is not within the remit of the KL-EC. Even if the allocation of applications to the individual medical specialties may be imprecise due to the limited options, the overall impression of the importance of the various medical specialties is well conveyed. A further aspect missing from our investigation is the costs associated with setting up and maintaining a qualified review board. Although the KL-EC members contribute voluntarily, office staff and premises, maintenance of software and servers and fees for external reviewers must be taken into account.

## Conclusion for practice

In Austria, in addition to the legally regulated ECs, voluntary institutional ECs make an important contribution to patient safety and the protection of vulnerable groups in research and to the advancement of medical research and education. Center-specific heterogeneity in composition, methods and decision making make harmonization through the development of common standards and operation procedures and mutual recognition desirable. The “Forum Österreichischer Ethikkommissionen” (Forum of Austrian ECs) might provide a suitable platform to achieve this goal.
